# A Data-Constrained and Physics-Guided Conditional Diffusion Model for Electrical Impedance Tomography Image Reconstruction

**DOI:** 10.3390/s26051728

**Published:** 2026-03-09

**Authors:** Xiaolei Zhang, Zhou Rong

**Affiliations:** College of Automation, Nanjing University of Posts and Telecommunications, Nanjing 210023, China; 1224056324@njupt.edu.cn

**Keywords:** conditional diffusion model, electrical impedance tomography, image reconstruction, deep learning, multi-source information fusion

## Abstract

**Highlights:**

- A multi-source conditional diffusion model is developed for electrical impedance tomography, enabling stable and accurate image reconstruction.
- A hybrid Swin–Mamba denoising network is introduced to efficiently capture both local structural details and global spatial consistency.
- The framework shows strong robustness and cross-system generalization across multiple real water tank platforms without retraining.
- The method enables noise-tolerant and high-resolution imaging for real-time medical and industrial sensing applications.

**Abstract:**

Electrical impedance tomography (EIT) provides noninvasive, high-temporal-resolution imaging for medical and industrial applications. However, accurate image reconstruction remains challenging due to the severe ill-posedness and nonlinearity of the inverse problem, as well as the limited robustness of existing single-source learning-based methods in real measurement scenarios. To address these limitations, a data-constrained and physics-guided Multi-Source Conditional Diffusion Model (MS-CDM) is proposed for EIT image reconstruction. Unlike conventional conditional diffusion methods that rely on a single measurement or an image prior, MS-CDM utilizes boundary voltage measurements as data-driven constraints and incorporates coarse reconstructions as physics-guided structural priors. This multi-source conditioning strategy provides complementary guidance during the reverse diffusion process, enabling balanced recovery of fine boundary details and global topological consistency. To support this framework, a Hybrid Swin–Mamba Denoising U-Net is developed, combining hierarchical window-based self-attention for local spatial modeling with bidirectional state-space modeling for efficient global dependency capture. Extensive experiments on simulated datasets and three real EIT experimental platforms demonstrate that MS-CDM consistently outperforms state-of-the-art numerical, supervised, and diffusion-based methods in terms of reconstruction accuracy, structural consistency, and noise robustness. Moreover, the proposed model exhibits robust cross-system applicability without system-specific retraining under multi-protocol training, highlighting its practical applicability in diverse real-world EIT scenarios.

## 1. Introduction

Electrical impedance tomography (EIT) is a noninvasive functional imaging technique that reconstructs the internal conductivity distribution of an object by injecting electrical currents through boundary electrodes and measuring the resulting voltage responses [1,2,3]. Owing to its radiation-free operation, low cost, fast response, and suitability for real-time monitoring, EIT has found wide applications in medical monitoring (such as pulmonary ventilation imaging [4] and early breast cancer detection [5]) and in industrial process tomography (including multiphase flow visualization [6] and robotic tactile sensing [7]). Compared with computed tomography (CT) and magnetic resonance imaging (MRI), EIT generally suffers from lower spatial resolution; however, its inherently high temporal resolution makes it uniquely advantageous for dynamic process monitoring [8].

Despite these benefits, accurate EIT image reconstruction remains a formidable challenge, as it constitutes a highly nonlinear and severely ill-posed inverse problem [4]. Due to the soft-field nature of EIT, boundary measurements exhibit low and nonuniform sensitivity to conductivity variations in the central region. Combined with measurement noise, modeling errors, and electrode contact impedance effects, reconstructed images often suffer from low resolution, blurred boundaries, and pronounced artifacts [9]. This pronounced sensitivity to measurement uncertainties significantly limits the accuracy and reliability of EIT in complex practical scenarios.

To mitigate these issues, conventional EIT reconstruction methods have primarily relied on linearization assumptions and regularization techniques. Early linear back-projection (LBP) algorithms offer computational simplicity but provide only qualitative imaging results [10]. The Gauss–Newton (GN) method iteratively approximates nonlinear solutions but is prone to convergence to local optima [11]. To alleviate ill-posedness, various regularization strategies have been introduced, including Tikhonov regularization [12], total variation (TV) regularization [13], and sparse Bayesian learning (SBL) [14]. Nevertheless, these approaches are highly sensitive to manually tuned hyperparameters: overly strong regularization leads to excessive smoothing and loss of structural details, whereas insufficient regularization fails to suppress noise. Moreover, such methods struggle to robustly handle complex and highly variable geometrical structures.

In recent years, deep learning has emerged as a data-driven paradigm for EIT reconstruction, enabling deep neural networks (DNN) to directly learn the nonlinear mapping from boundary voltages to conductivity distributions [15,16,17]. Tan et al. demonstrated the feasibility of convolutional neural networks (CNN) for EIT imaging [18]. Hamilton et al. combined U-Net architectures with D-bar methods to enhance denoising performance [19]. Wei et al. introduced dominant current features to improve corner detection [20], while Chen et al. proposed a structure-aware dual-branch network (SADB-Net) to refine detail reconstruction [21]. Li et al. further employed generative adversarial networks (GAN) with soft attention mechanisms to enhance texture recovery [22] and extended U-Net-based semantic segmentation to three-dimensional pneumothorax volume assessment [23]. Although these DL-based approaches outperform traditional algorithms, most of them rely on deterministic regression, which is susceptible to overfitting. In addition, the lack of explicit physical constraints often limits their generalization capability when transferring from simulated environments to real experimental settings.

As a class of generative models, diffusion probabilistic models (DPM) have recently provided a new perspective for addressing the ill-posed nature of EIT reconstruction [24,25]. Liu et al. proposed DiffusionEIT, which integrates voltage data via a cross-modal attention Transformer to achieve high-resolution reconstruction [26]. Shi et al. developed CDEIT using a Transformer-based U-Net to implicitly learn conductivity priors [27]. These studies mark a paradigm shift from deterministic regression to generative modeling in EIT, demonstrating the strong potential of DPM in noise suppression and fine-detail recovery.

Nevertheless, existing diffusion-based EIT methods still face notable limitations. First, most approaches rely on a single conditioning source (either boundary voltages or preliminary reconstruction images), thereby failing to jointly exploit the complementary strengths of precise physical constraints and spatial structural priors. Second, physical consistency is often weakly enforced, resulting in visually plausible images that may violate the underlying EIT forward model. Finally, domain shift caused by electrode contact impedance and hardware discrepancies remains a critical challenge, hindering robust generalization from simulation to real-world measurements. To address these challenges, this study proposes a data-constrained and physics-guided Multi-Source Conditional Diffusion Model (MS-CDM) for EIT image reconstruction. The main contributions of this work are summarized as follows:

(1) A physics-guided and data-constrained multi-source conditional diffusion framework is introduced for EIT reconstruction, which jointly exploits boundary voltage measurements as data-driven constraints and GN reconstructions as physics-informed structural priors, thereby effectively mitigating the ill-posedness of the EIT inverse problem.
(2) A Hybrid Swin–Mamba Denoising U-Net is developed as the diffusion backbone, combining hierarchical window-based self-attention for local spatial modeling with bidirectional state-space modeling to efficiently capture long-range dependencies, leading to improved boundary delineation and global topological consistency.
(3) A multi-source conditional fusion strategy is incorporated into the reverse diffusion process, enabling complementary guidance from measurement-domain and image-domain priors, which substantially enhances reconstruction accuracy, noise robustness, and structural stability compared with single-source diffusion approaches.
(4) Comprehensive evaluations on simulated datasets and multiple real EIT platforms validate the effectiveness and generalization capability of the proposed method, demonstrating consistent performance gains over state-of-the-art numerical, supervised, and diffusion-based reconstruction techniques without system-specific retraining.

## 2. Problem Formulation

### 2.1. EIT Forward Problem Modeling

Under the quasi-static approximation, the physical process of EIT can be simplified from Maxwell’s equations to an elliptic partial differential equation. Let $\Omega \subset R^{2}$ denote the imaging domain. The internal electric potential u(r) and the conductivity distribution σ(r) satisfy the following governing equation:

(1) ∇⋅(σ(r)∇u(r))=0,r∈Ω

where r denotes the spatial position vector.

**Figure 1:**
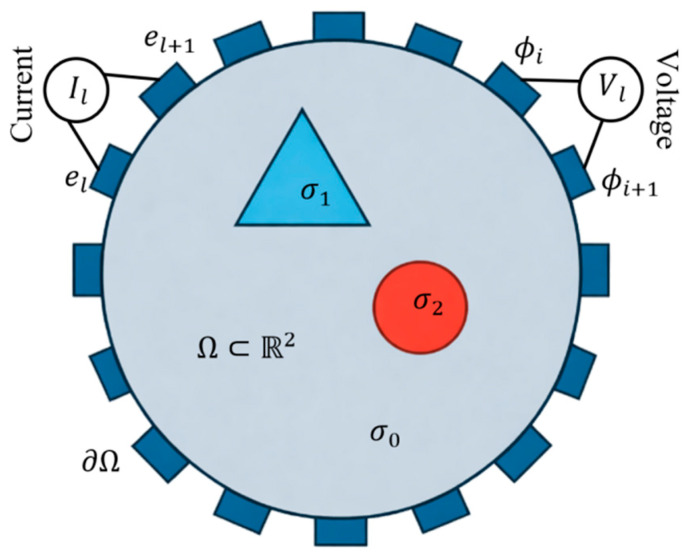
Schematic illustration of the two-dimensional EIT forward model with 16 uniformly distributed boundary electrodes.

As illustrated in Figure 1, the complete electrode model (CEM) is adopted to describe the EIT forward problem [28]. Let $\{E_{l}{\}}_{l=1}^{L}$ denote L=16 electrodes uniformly distributed on the boundary ∂Ω. The boundary conditions are given by:

(2) $$\left\{\begin{array}{ll} u+z_{l}\sigma \frac{\partial u}{\partial n}=U_{l}, & \mathrm{on}\;e_{l}\\ \int_{e_{l}}\;\;\sigma \frac{\partial u}{\partial n}ds=I_{l}, & l=1,\ldots ,L\\ \sigma \frac{\partial u}{\partial n}=0, & \mathrm{on}\;\partial \Omega \setminus \overset{L}{\underset{l=1}{⋃}}\;\;e_{l} \end{array}\right.$$

where $e_{l}$ denotes the *l*-th electrode, $z_{l}$ is the corresponding contact impedance, *n* represents the outward unit normal vector on the boundary, and $U_{l}$ and $I_{l}$ denote the boundary voltage and injected current at the l-th electrode, respectively.

To mitigate the influence of system errors, inter-subject variability, and uncertainties in electrode contact impedance, a time-difference EIT imaging strategy is employed [29,30]. Let $\sigma_{1}$ and $\sigma_{2}$ denote the absolute conductivity distributions at the reference time and the current time, respectively. The reconstruction target is defined as the conductivity change:

(3) $$\Delta \sigma =\sigma_{2}-\sigma_{1}$$

Accordingly, the boundary voltage difference is defined as:

(4) $$\Delta v=v_{2}-v_{1}$$

where $v_{1}=F(\sigma_{1})$ and $v_{2}=F(\sigma_{2})$ are boundary voltages generated by the nonlinear EIT forward operator F(⋅). Therefore, the time-difference EIT forward relationship can be written as:

(5) $$\Delta v=F(\sigma_{2})-F(\sigma_{1})=F(\sigma_{1}+\Delta \sigma )-F(\sigma_{1})$$

For practical reconstruction, a first-order linearization around the reference state $\sigma_{1}$ is commonly adopted, yielding

(6) $$\Delta v\approx J(\sigma_{1})\;\Delta \sigma$$

where $J\left(\sigma_{1}\right)$ denotes the Jacobian (sensitivity) matrix of the forward operator evaluated at $\sigma_{1}$.

### 2.2. EIT Inverse Problem

The EIT inverse problem aims to reconstruct the internal conductivity distribution **σ** from the measured boundary voltage data **v**. Due to the strong nonlinearity of the operator F(⋅) and the presence of measurement noise, this inverse problem is severely ill-posed. Within a deep learning framework [15], the inverse problem is commonly formulated as a supervised optimization task, with the objective function expressed as:

(7) $$\sigma^{*}=arg\;\underset{\sigma }{min}\;\left(\underset{L_{data}}{\underbrace{\|\sigma_{0}-\sigma {\|}_{2}^{2}}}+\lambda \underset{L_{phys}}{\underbrace{\|v-F(\sigma ){\|}_{2}^{2}}}\right)$$

where $L_{data}$ denotes the data-consistency term that enforces the reconstructed image to approximate the ground-truth conductivity distribution $\sigma_{0}$, $L_{phys}$ represents the physics-consistency term that constrains the solution to satisfy the EIT forward model, and λ is a balancing coefficient.

Different from the above deterministic point-estimation paradigm, the proposed Multi-Source Conditional Diffusion Model (MS-CDM) reformulates the EIT reconstruction task as a conditional probability learning problem. Specifically, the measured boundary voltage $y_{Vol}\in R^{208\times 1}$ (data constraint) and the reconstructed image $y_{GN}\in R^{64\times 64}$ (physics-guided prior) are jointly introduced as dual conditions C. The model learns the posterior distribution of the true conductivity distribution as:

(8) $$p\left(\sigma_{0}|C\right),\;C=\{y_{Vol},y_{GN}\}$$

In this manner, high-resolution conductivity reconstruction can be achieved while preserving the stability of the underlying physical structure.

## 3. Multi-Source Conditional Diffusion Model

### 3.1. Forward Diffusion Process

Within the diffusion model framework [31], the forward diffusion process gradually perturbs the ground-truth conductivity distribution $\sigma_{0}$ by injecting Gaussian noise, thereby mapping it to a sequence of noisy state $\{\sigma_{t}{\}}_{t=1}^{T}$. This process is defined as:

(9) $$q(\sigma_{t}|\sigma_{0})=N(\sigma_{t};\sqrt{{\overset{-}{\alpha }}_{t}}\sigma_{0},(1-{\overset{-}{\alpha }}_{t})I)$$

where $\{\beta_{t}{\}}_{t=1}^{T}$ denotes a predefined noise schedule; $\alpha_{t}=1-\beta_{t}$, ${\overset{-}{\alpha }}_{t}=\prod_{i=1}^{t}\alpha_{i}$, and I represent the identity matrix.

Exploiting the reparameterization property of Gaussian distributions, Equation (8) can be equivalently expressed as

(10) $$\sigma_{t}=\sqrt{{\overset{-}{\alpha }}_{t}}\;\sigma_{0}+\sqrt{1-{\overset{-}{\alpha }}_{t}}\;\varepsilon ,\;\;\;\;\;\varepsilon ∼N(0,I)$$

which indicates that, as the diffusion step *t* increases, the spatial structural information in the original conductivity image is progressively corrupted by noise and eventually converges to a standard Gaussian distribution.

### 3.2. Conditional Reverse Process

The objective of the reverse diffusion process is to progressively recover the original conductivity distribution $\sigma_{0}$ from the noisy state $\sigma_{t}$, under the guidance of the multi-source condition C. This process is implemented by learning the conditional transition probability:

(11) $$p_{\theta }(\sigma_{t-1}|\sigma_{t},C)$$

where θ denotes the network parameters. In practice, a neural network $\epsilon_{\theta }(\sigma_{t},t,C)$ is employed to directly predict the noise component introduced at the current diffusion step. Based on the predicted noise, the original conductivity distribution can be analytically estimated as:

(12) $${\hat{\sigma }}_{0}=\frac{1}{\sqrt{{\overset{-}{\alpha }}_{t}}}\left(\sigma_{t}-\sqrt{1-{\overset{-}{\alpha }}_{t}}\;\epsilon_{\theta }(\sigma_{t},t,C)\right)$$

Unlike diffusion-based EIT reconstruction methods that rely on a single conditioning source, the proposed MS-CDM simultaneously incorporates data-driven constraints (boundary voltage measurements) and physics-guided priors (reconstructed images). These dual constraints jointly regulate the denoising trajectory during the reverse diffusion process, thereby substantially improving reconstruction stability and physical consistency.

### 3.3. Physics-Enhanced Loss

To balance reconstruction accuracy and physical consistency, a physics-enhanced composite loss function is formulated as:

(13) $$L_{total}=L_{data}+\gamma L_{phys}$$

where $L_{data}$ denotes the data-driven denoising loss, defined as:

(14) $$L_{denoise}=E_{\sigma_{0},\epsilon ,t}[\|\epsilon -\epsilon_{\theta }(\sigma_{t},t,C){\|}_{2}^{2}]$$

and $L_{phys}$ enforces consistency between the generated conductivity image and the actual measurements through the forward physical operator:

(15) $$L_{phys}=E_{\sigma_{0}}[\|F({\hat{\sigma }}_{0})-y_{Vol}{\|}_{2}^{2}]$$

where ${\hat{\sigma }}_{0}$ is derived from the current noisy state $\sigma_{t}$ and predicted noise $\epsilon_{\theta }$. The weighting coefficient γ enables dynamic balancing between physics-based constraints and data-driven learning.

### 3.4. Hybrid Swin–Mamba Denoising U-Net

**Figure 2:**
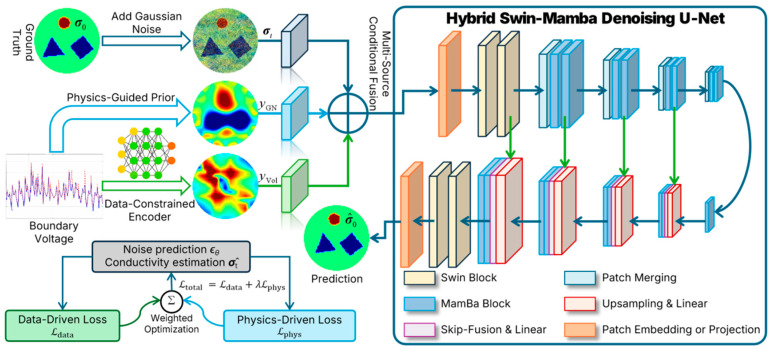
Overall framework of the proposed MS-CDM. Boundary voltage measurements are integrated as data-driven constraints, while physics-guided priors serve as structural anchors to jointly modulate a Hybrid Swin–Mamba Denoising U-Net. The model iteratively reconstructs the conductivity distribution by predicting the noise term at each diffusion step. The training objective employs a composite loss function that balances denoising accuracy with physics-driven data consistency.

To enhance reconstruction accuracy and stability in EIT inverse problems, a multi-source condition–driven hybrid denoising network is proposed, as illustrated in Figure 2. The network adopts a U-Net architecture as the overall backbone and integrates complementary feature modeling modules at different hierarchical levels. Specifically, Swin Transformer blocks are embedded in shallow and intermediate layers to strengthen local texture and boundary detail modeling, while bidirectional Mamba state-space modules (Bi-Mamba) are incorporated in deep layers to efficiently capture global structural dependencies. In addition, gated feature fusion units are employed to enable cooperative integration of heterogeneous features. This design jointly balances physical constraints, hierarchical feature representation, computational efficiency, and engineering feasibility.

#### 3.4.1. Multi-Source Condition Encoding and Fusion

One of the fundamental challenges in EIT inverse problems lies in the strong heterogeneity between measurement-domain signals and image-domain targets in both dimensionality and structure. To address this issue, a dual-branch conditional encoder is designed to map boundary voltage measurements and physics-guided priors into a unified conditional tensor space aligned with image features, enabling effective cross-domain fusion.

In data-constrained encoding, the measured boundary voltage $v_{\mathrm{meas}}$ contains essential boundary response information. A multi-layer perceptron (MLP) is employed to perform nonlinear projection, followed by a reshape operation to transform the high-dimensional features into spatially aligned two-dimensional conditional maps:

(16) $$y_{\mathrm{Vol}}\;=\;\mathrm{Reshape}(\mathrm{MLP}(v_{\mathrm{meas}}))$$

This spatialization allows measurement-domain information to participate in the diffusion denoising process at the pixel level, thereby continuously enforcing data consistency during image generation.

In physics-guided prior, to reduce the search space of the generative model and enhance topological stability, a one-step GN image reconstruction is introduced as a physics-guided prior [32]:

(17) $$y_{GN}=(J^{T}J+\lambda R{)}^{-1}J^{T}(v_{\mathrm{meas}}-F(\sigma_{ref}))$$

where J denotes the sensitivity matrix, R is the Tikhonov regularization matrix, F(⋅) represents the EIT forward operator, and $\sigma_{ref}$ is the reference conductivity distribution. This prior exhibits high spatial alignment with the target conductivity image and provides stable structural guidance, effectively suppressing nonphysical artifacts caused by noise and ill-posedness.

#### 3.4.2. Hierarchical Spatial Modeling via Swin Transformer

**Figure 3:**
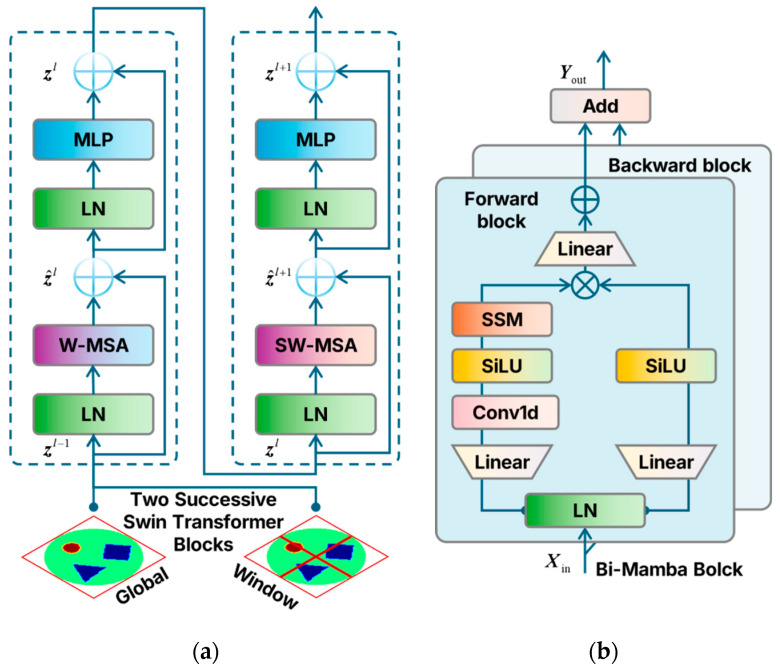
Architecture of the hybrid Swin–Mamba denoising U-Net. (**a**) Swin Transformer blocks deployed in shallow and intermediate layers for hierarchical spatial modeling using window-based and shifted-window self-attention. (**b**) Bi-Mamba blocks integrated into deep encoder layers to perform bidirectional state-space modeling for efficient capture of global structural dependencies.

To enhance spatial structure representation while maintaining computational efficiency, Swin Transformer blocks are incorporated into the shallow and intermediate layers of the denoising U-Net, as shown in Figure 3a. The core idea is to compute self-attention within local windows (Window-based Multi-head Self-Attention, W-MSA) and introduce shifted windows (Shifted Window MSA, SW-MSA) in adjacent layers to enable cross-window information exchange, thereby constructing hierarchical spatial modeling from local to global scales.

Let the input feature map be:

(18) $$Z\in R^{H\times W\times D}$$

where *H* and *W* denote the spatial dimensions and *D* denotes the channel dimension. The feature map is partitioned into *N_w_* non-overlapping windows of size M×M, yielding token sequences:

(19) $$X\in R^{M^{2}\times D}$$

Linear projections for multi-head attention are given by:

(20) $$Q=XW_{Q},\;\;\;\;\;\;\;K=XW_{K},\;\;\;\;\;\;\;V=XW_{V}$$

where $W_{Q},W_{K},W_{V}\in R^{D\times d}$. The window-based attention is computed as:

(21) $$Attention(Q,K,V)=Softmax\left(\frac{QK^{⊤}}{\sqrt{d_{k}}}+B\right)V$$

where $d_{k}$ is the scaling factor and $B\in R^{M^{2}\times M^{2}}$ is the relative position bias matrix.

To alleviate window-wise isolation, shifted window attention is applied in adjacent blocks. Two consecutive Swin blocks can be expressed as:

(22) $$Z_{1}^{l}=W-MSA(LN(Z^{l}))+Z^{l}$$

(23) $$Z^{l+1}=MLP(LN(Z_{1}^{l}))+Z_{1}^{l}$$

(24) $$Z_{1}^{l+1}=SW-MSA(LN(Z^{l+1}))+Z^{l+1}$$

(25) $$Z^{l+2}=MLP(LN(Z_{1}^{l+1}))+Z_{1}^{l+1}$$

where LN(⋅) denotes layer normalization, and the MLP is defined as:

(26) $$MLP(u)=W_{2}\;\phi (W_{1}u)$$

with ϕ(⋅) being the activation function.

#### 3.4.3. Global State-Space Modeling via Bi-Mamba

Although Swin Transformer blocks effectively model local and mid-range spatial structures, deeper layers of the U-Net (with lower resolution and higher semantic abstraction) require stronger global dependency modeling. To address the computational and memory limitations of Transformers in this regime, bidirectional Mamba state-space modules (Bi-Mamba) are introduced in the deep encoder layers, as illustrated in Figure 3b.

The deep feature maps are flattened or organized as sequences:

(27) $$x_{t}\in R^{D}$$

The continuous-time state-space model is defined as:

(28) $$\frac{dh(t)}{dt}=Ah\left(t\right)+Bx\left(t\right),\;\;\;\;\;\;\;y(t)=Ch(t)+Dx(t)$$

which is discretized into the recursive form:

(29) $$h_{t}=\overset{-}{A}h_{t-1}+\overset{-}{B}x_{t},y_{t}=Ch_{t}+Dx_{t}$$

where $h_{t}\in R^{N}$ denotes the latent state vector and A,B,C,D are learnable parameters.

To capture bidirectional contextual information, Bi-Mamba performs parallel forward and backward scans:

(30) $$Y_{\to }={SSM}_{\to }\left(X\right),\;\;\;\;\;\;\;Y_{\leftarrow }={SSM}_{\leftarrow }(X)$$

which are concatenated along the channel dimension to form:

(31) $$Y_{out}=Concat(Y_{\to },Y_{\leftarrow })$$

Here, this structure captures global structural dependencies with near-linear complexity, enabling robust recovery of large-scale continuous boundaries and low-frequency topological patterns in EIT.

### 3.5. Sampling Strategy for Fast Imaging

During inference, a denoising diffusion implicit model (DDIM) is adopted to accelerate sampling. The update rule is given by:

(32) $$\sigma_{t-1}=\sqrt{{\overset{-}{\alpha }}_{t-1}}{\hat{\sigma }}_{0}(\sigma_{t})+\sqrt{1-{\overset{-}{\alpha }}_{t-1}}\cdot \epsilon_{\theta }(\sigma_{t},t,C)$$

This strategy significantly reduces the number of sampling steps (e.g., to 50 steps) while preserving reconstruction quality, thereby enabling MS-CDM to meet the practical requirements of sensor applications such as real-time biomedical imaging and industrial monitoring.

The training procedure of the proposed MS-CDM is summarized in Algorithm 1. Specifically, the model is optimized by minimizing a physics-enhanced composite loss that jointly enforces denoising accuracy and EIT data consistency. During inference, the conductivity distribution is reconstructed by iteratively applying the conditional reverse denoising process, as outlined in Algorithm 2.
**Algorithm 1** Training Algorithm of the Proposed MS-CDM**Input:** Paired training data $\left(y_{vol},y_{GN},\sigma_{0}\right)$; Noise schedule: $\left\{\beta_{1},\beta_{2},\ldots ,\beta_{T}\right\}$; EIT forward operator F(·). **Output:** Trained model parameters θ 1. Initialize network parameters θ randomly; 2. **repeat** 3.    Sample Gaussian noise ε∼N(0,I); 4.    Sample diffusion step t∼U({1,2,…,T}); 5.    Generate the noisy conductivity image according to the forward diffusion process:         $\sigma_{t}=\sqrt{{\overset{-}{\alpha }}_{t}}\;\sigma_{0}+\sqrt{1-{\overset{-}{\alpha }}_{t}}\;\varepsilon$; 6.    Predict the noise term using the conditional denoising network:         $\hat{\epsilon }=\epsilon_{\theta }\left(\sigma_{t}|y_{vol},y_{GN}\right)$; 7.    Compute the denoised conductivity estimate ${\hat{\sigma }}_{0}$ using Equation (11); 8.    Compute the total loss using the physics-enhanced objective:         $L=L_{denoise}+\lambda L_{phys}$; 9.    Update network parameters θ using the Adam optimizer; 10. **until** the training phase ends.

**Algorithm 2** Reconstruction (Inference) of the Proposed MS-CDM**Input:** Test conditions $\left(y_{vol},y_{GN}\right)$; Noise schedule $\left\{\beta_{1},\beta_{2},\ldots ,\beta_{T}\right\}$; Total diffusion steps *T*. **Output:** Reconstructed conductivity distribution ${\hat{\sigma }}_{0}$. 1. Initialize $\sigma_{T}∼N(0,I)$; 2. **for**
t=T,T−1,…,1 **do** 3.    Predict the noise term:         $\hat{\epsilon }=\epsilon_{\theta }\left(\sigma_{t}|y_{vol},y_{GN}\right)$; 4.    Estimate the denoised conductivity ${\hat{\sigma }}_{0}$ using Equation (11); 5.    Sample $\sigma_{t-1}$ from the DDIM sampler according to Equation (32); 6. **end for** 7. Output ${\hat{\sigma }}_{0}$.

## 4. Experimental Setup

### 4.1. Simulated Dataset Construction

**Figure 4:**
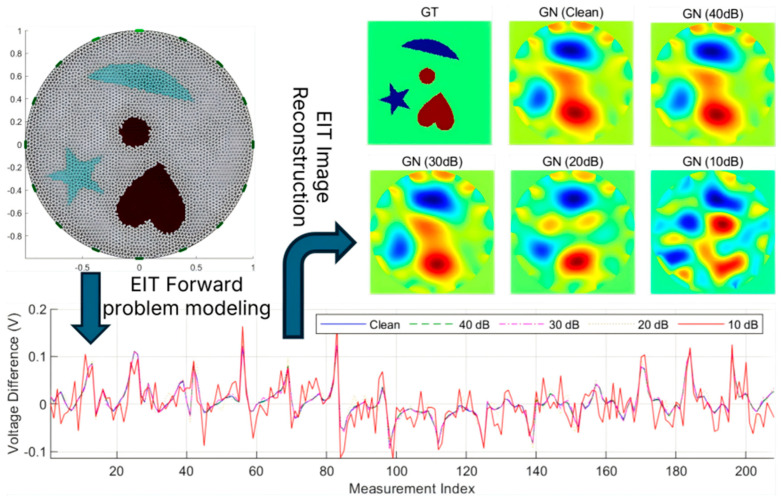
Workflow of the simulated EIT dataset construction.

A simulated dataset was constructed based on the open-source EIDORS toolbox to alleviate data scarcity and improve the generalization ability of deep learning models for EIT inverse problems [33]. The overall simulation pipeline is illustrated in Figure 4, and the detailed configuration is described as follows.

To approximate realistic physical field distributions and enhance adaptability across different EIT hardware systems, the EIT forward problem was numerically solved using the two-dimensional finite element method (FEM). A high-density adaptive triangular mesh was employed for forward computation. Each frame was generated using 16 uniformly distributed boundary electrodes under the adjacent current injection–adjacent voltage measurement protocol, yielding 208 independent boundary voltage measurements per frame.

To cover heterogeneous hardware specifications, three excitation current protocols were simulated: the UEF standard mode (1.0 mA) [34], the KTC rotating electrode mode (2.0 mA) [35], and a custom EIT system mode (2.5 mA). This multi-protocol data generation strategy substantially increases the physical diversity of the dataset.

**Figure 5:**
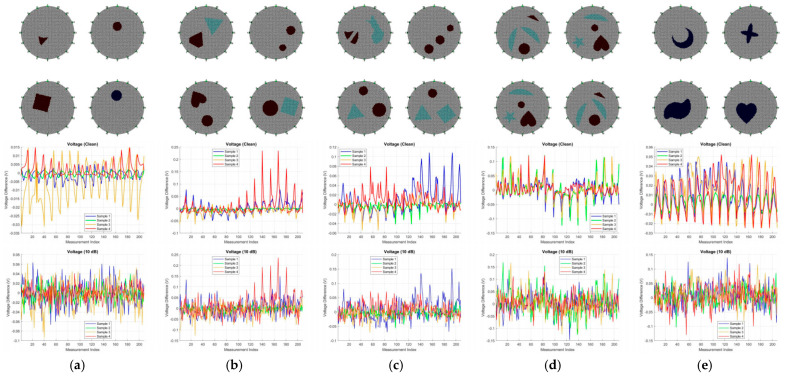
Boundary voltage measurements under different target configurations. (**a**) Single target; (**b**) Two targets; (**c**) Three targets; (**d**) Four targets; (**e**) Concave target.

The background conductivity was fixed at 1.0 S/m. To simulate complex two-phase flow patterns and heterogeneous biological tissues, five subsets were generated, including Single, Two, Three, Four, and Complex target configurations, as shown in Figure 5. The inclusions covered both basic geometric shapes (e.g., circles, triangles, and squares) and complex non-convex shapes (e.g., pentagrams, hearts, and crescents), enhancing the model’s sensitivity to diverse topological patterns. To emulate dynamic physical behaviors, two random motion augmentation modes were designed: (a) Independent motion, where each target undergoes random translation and rotation independently; (b) global motion, where all targets preserve relative positions while undergoing collective translation and rotation. Regarding conductivity properties, inclusions were randomly assigned as either low-conductivity phases (0.1 S/m) or high-conductivity phases (10.0 S/m), with an additional 10% random amplitude perturbation applied to simulate material inhomogeneity.

To enhance the robustness of MS-CDM under low signal-to-noise ratio (SNR) conditions, a Noise-to-Clean supervised learning strategy was adopted. Specifically, Gaussian white noise of varying intensities was added to noise-free boundary voltage measurements $v_{\mathrm{clean}}$ to simulate electronic noise and environmental disturbances. According to

(33) $${SNR}_{dB}=10{log}_{10}(\frac{P_{signal}}{P_{noise}})$$

noisy voltage samples were generated at five SNR levels: Inf (noise-free), 40 dB, 30 dB, 20 dB, and 10 dB. The first model input was defined as the normalized boundary voltage difference vector $y_{\mathrm{noise}}$, computed as

(34) $$y_{noise}=v_{noisy}-v_{ref}$$

where $v_{\mathrm{noisy}}$ denotes the noisy measurement at the current time and $v_{\mathrm{ref}}$ is the reference voltage under homogeneous conditions.

A total of 50,000 simulated samples were generated and evenly distributed across the five subsets (10,000 samples per subset). All conductivity images were initially generated at a resolution of 256 × 256 and then interpolated to 64 × 64 to match the network input. Five-fold cross-validation was employed to evaluate generalization performance and prevent overfitting. The dataset was randomly partitioned into five mutually exclusive subsets, with 80% used for training and 20% for validation, and the final results were averaged over all five folds.

### 4.2. Evaluation Metrics

All models were implemented using the PyTorch (version 1.12.1) framework and trained on a high-performance workstation equipped with an Intel Core i9-13900K CPU and an NVIDIA GeForce RTX 4090 GPU. The proposed MS-CDM was optimized using the AdamW optimizer with an initial learning rate of 0.001, followed by a cosine annealing schedule. The batch size was set to 32, and the model was trained for 300 epochs. During inference, a DDIM sampler was employed with 200 sampling steps to accelerate reconstruction.

To quantitatively evaluate reconstruction quality, four widely used metrics were adopted. Relative Error (RE) quantifies the overall numerical deviation between reconstructed and ground-truth images. Correlation Coefficient (CC) evaluates the linear correlation between reconstructed and true conductivity distributions, reflecting positional and morphological consistency. Structural Similarity Index (SSIM) assesses perceptual similarity in terms of luminance, contrast, and structure, ranging from 0 to 1. Dice Similarity Coefficient (Dice) measures the spatial overlap between reconstructed and ground-truth conductivity distributions, with values closer to 1 indicating higher structural agreement.

The corresponding definitions are given by:

(35) $$\mathrm{RE}=\frac{\|\overset{ˆ}{x}-x{\|}_{2}}{\|x{\|}_{2}}=\frac{\sqrt{\sum_{i=1}^{N}({\overset{ˆ}{x}}_{i}-x_{i}{)}^{2}\;}}{\sqrt{\sum_{i=1}^{N}x_{i}^{2}}}$$

(36) $$\mathrm{CC}=\frac{\sum_{i=1}^{N}\left(x_{i}-\overset{‾}{x})({\overset{ˆ}{x}}_{i}-\bar{\overset{ˆ}{x}}\right)}{\sqrt{\sum_{i=1}^{N}(x_{i}-\overset{‾}{x}{)}^{2}\;}\cdot \sqrt{\sum_{i=1}^{N}\;({\overset{ˆ}{x}}_{i}-\bar{\overset{ˆ}{x}}{)}^{2}}}$$

(37) $$\mathrm{SSIM}(x,\overset{ˆ}{x})=\frac{\left(2\mu_{x}\mu_{\overset{ˆ}{x}}+C_{1})(2\sigma_{x\overset{ˆ}{x}}+C_{2}\right)}{\left(\mu_{x}^{2}+\mu_{\overset{ˆ}{x}}^{2}+C_{1})(\sigma_{x}^{2}+\sigma_{\overset{ˆ}{x}}^{2}+C_{2}\right)}$$

(38) $$Dice=\frac{2\;|x\cap \hat{x}|}{\left|x|+|\hat{x}\right|}=\frac{2\sum_{i=1}^{N}x_{i}{\hat{x}}_{i}}{\sum_{i=1}^{N}x_{i}+{\sum_{i=1}^{N}\hat{x}}_{i}}$$

where x and $\overset{ˆ}{x}$ denote the ground-truth and reconstructed conductivity images, respectively, and *N* is the total number of pixels. μ, σ denote mean and variance, $C_{1}$ and $C_{2}$ are stability constants, and $\|\cdot {\|}_{2}$ denotes the $L_{2}$ norm.

For real phantom-based experimental data, a true pixel-wise internal conductivity distribution is not directly available. Therefore, the quantitative metrics are computed with respect to a reference map constructed from the known phantom design, rather than a true physical ground truth. Specifically, the background, low-conductivity inclusion, and high-conductivity inclusion are encoded as 0, 1, and 2, respectively, to form a standardized reference map for evaluation.

This region-wise encoding does not represent the exact continuous conductivity distribution, and thus the resulting metrics should be interpreted as reference-based comparative metrics rather than absolute reconstruction accuracy. Accordingly, qualitative visual assessment (e.g., shape fidelity, boundary localization, and artifact suppression) is also used as an important part of the evaluation.

### 4.3. Comparison Methods

To comprehensively evaluate the performance of MS-CDM in terms of reconstruction accuracy, noise robustness, and generalization capability, three categories of representative EIT reconstruction methods were selected for comparison under identical experimental conditions.

The TR (traditional regularized reconstruction) method was used as the representative conventional numerical baseline. In this work, TR was implemented using a GN reconstruction framework, in which the NOSER (Newton One-Step Error Reconstructor [36]) prior was adopted as the regularization operator, with the regularization parameter set to λ=0.05. Although this regularized GN-based reconstruction can partially address nonlinearity through least-squares optimization, it often suffers from excessive smoothing and blurred boundaries. Since the same type of TR reconstruction is also used to generate the physics-guided prior in MS-CDM, this baseline enables direct quantification of the performance gains achieved by the proposed framework.

Among supervised deep learning methods, a conventional stacked CNN (Convolutional Neural Network) was included, which directly learns the nonlinear mapping from boundary voltages to conductivity distributions and represents an early end-to-end imaging paradigm [37]. In addition, RAU-Net (Residual Attention U-Net) was selected, which integrates residual connections and attention mechanisms to emphasize salient features and mitigate gradient degradation [23]. Furthermore, DHU-Net (Dual-Branch U-Net), designed for EIT soft-field effects, was included; it incorporates deformable convolutions to handle irregular target shapes and squeeze-and-excitation attention for enhanced global feature extraction [38].

CDEIT (Conditional Diffusion Model for EIT reconstruction) represents a state-of-the-art diffusion-based EIT reconstruction approach that conditions the reverse denoising process solely on boundary voltage measurements [27]. Comparing MS-CDM with CDEIT specifically highlights the effectiveness of the proposed dual-conditioning mechanism, which combines physics-guided priors with data-driven constraints to improve geometric stability and artifact suppression.

All deep learning models (CNN, RAU-Net, DHU-Net, and CDEIT) were reimplemented using the PyTorch framework. The network architectures and hyperparameters were set by following the original publications as closely as possible. All models were trained using the Adam optimizer on identical training and testing datasets until convergence, ensuring an objective and fair comparison.

## 5. Simulation Results and Analysis

### 5.1. Comparison of Multiphase Conductivity Reconstruction on Simulated Data

To systematically evaluate the reconstruction performance of different EIT methods under controlled conditions, extensive comparative experiments were conducted on the simulated multiphase conductivity dataset. Owing to the availability of ground-truth conductivity distributions in simulation, both quantitative evaluation metrics and qualitative visualizations were employed to compare traditional numerical methods, supervised deep learning approaches, and diffusion-based generative models. Table 1 summarizes the quantitative reconstruction performance of all comparison methods on the simulated test dataset, evaluated using RE, CC, SSIM, and Dice.

Overall, the traditional TR method exhibits the poorest performance across all metrics, with a high relative error of 0.952 ± 0.303 and substantially low SSIM and Dice values, indicating severe deficiencies in both numerical accuracy and structural fidelity. This outcome is expected, as TR is a single-step linear reconstruction approach that lacks nonlinear modeling capability and effective spatial regularization. Supervised CNN-based methods yield notable improvements over TR, reducing the relative error to 0.564 ± 0.108 and achieving moderate gains in CC and SSIM. However, structural consistency remains limited, with a Dice score of only 0.756 ± 0.100, primarily due to the absence of explicit physical constraints and global structural priors. By incorporating residual connections and attention mechanisms, RAU-Net further enhances reconstruction quality, achieving an SSIM of 0.885 ± 0.058 and a Dice score of 0.871 ± 0.139. Nevertheless, its relative error remains comparatively high with larger variance, suggesting insufficient robustness when handling complex or multi-target conductivity distributions. Among supervised learning approaches, DHU-Net demonstrates superior performance, benefiting from its dual-branch architecture tailored to the soft-field nature of EIT. The relative error is reduced to 0.337 ± 0.110, and the Dice coefficient increases to 0.901 ± 0.060, indicating improved reconstruction accuracy and structural preservation. Despite these gains, DHU-Net remains a deterministic regression model and does not explicitly account for the inherent uncertainty of the EIT inverse problem.

**Figure 6:**
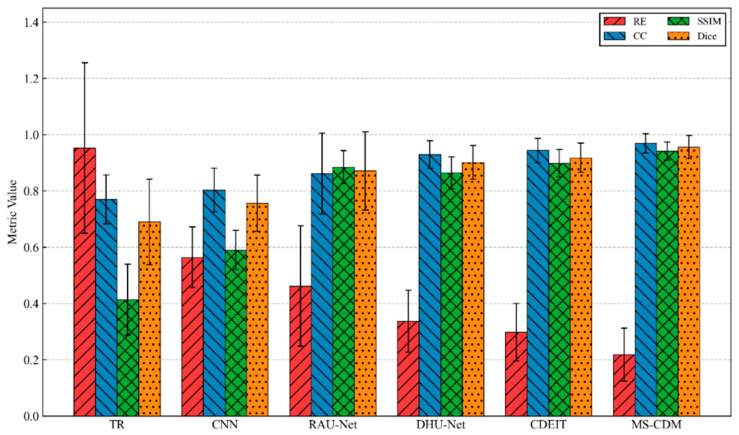
Quantitative comparison of different EIT reconstruction methods on the simulated dataset using four evaluation metrics.

In the category of generative methods, CDEIT consistently outperforms traditional and supervised models, achieving further improvements across all evaluation metrics. This result confirms the effectiveness of diffusion-based iterative denoising for modeling complex conductivity distributions. However, since CDEIT relies solely on boundary voltage measurements as conditioning information, its utilization of physical priors remains limited. The proposed MS-CDM achieves the best performance across all quantitative metrics, with the lowest relative error (0.218 ± 0.094), highest correlation coefficient (0.969 ± 0.034), and a Dice score of 0.956 ± 0.041, as also illustrated in Figure 6. Compared with the second-best method, CDEIT, MS-CDM exhibits clear advantages in both numerical accuracy and structural consistency, while also yielding smaller standard deviations, indicating more stable reconstruction performance across diverse test samples. These results quantitatively validate the effectiveness of integrating physics-guided priors and data-constrained conditions within the diffusion framework.

In addition to reconstruction accuracy, computational efficiency was also considered for practical applicability. Preliminary comparisons of DDIM sampling with 50, 100, 150, and 200 steps showed that the quantitative performance differences were relatively small, whereas the inference time increased substantially with the number of sampling steps. Therefore, DDIM with 50 sampling steps was adopted in this work as the default setting to achieve a better trade-off between reconstruction quality and computational cost. Specifically, the average per-image inference times of MS-CDM were 245.2 ms, 488.5 ms, 731.1 ms, and 975.8 ms for DDIM-50, DDIM-100, DDIM-150, and DDIM-200, respectively, measured under the same hardware/software environment.

Under the same hardware/software environment, the per-image inference time of MS-CDM (DDIM-50) was further compared with the baseline methods (GN, CNN, DHU-Net, and CDEIT). The average per-image inference times were 9.4 ms for GN, 18.7 ms for CNN, 42.6 ms for DHU-Net, 180.7 ms for CDEIT, and 245.2 ms for MS-CDM (DDIM-50). These results show that MS-CDM requires more computation than conventional deterministic reconstruction methods and lightweight supervised networks due to iterative diffusion sampling, but remains substantially more efficient than higher-step diffusion settings while providing superior reconstruction quality. This observation highlights the practical value of the proposed multi-source conditioning strategy and supports its potential applicability in time-sensitive EIT monitoring scenarios, subject to the specific latency requirements of the target application.

**Figure 7:**
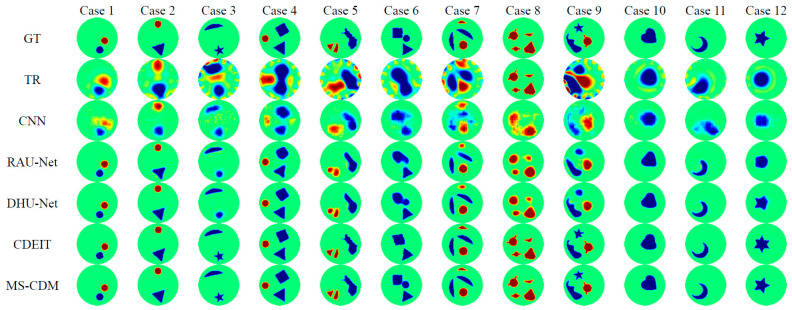
Qualitative comparison of different EIT reconstruction methods on the simulated dataset. Each column corresponds to one test sample (Case 1–Case 12). The first row shows the ground-truth conductivity distributions (GT), followed by reconstruction results obtained using TR, CNN, RAU-Net, DHU-Net, CDEIT, and the proposed MS-CDM, respectively.

Representative qualitative reconstruction results from the simulated test dataset are presented in Figure 7, covering conductivity scenarios of increasing complexity. TR fails to localize conductivity inclusions in most cases, producing overly smoothed and spatially distorted reconstructions. CNN-based methods improve target localization but suffer from blurred boundaries and shape distortions in multi-target or complex scenarios. RAU-Net yields clearer edges but still exhibits shape inaccuracies and intensity inconsistencies under increased complexity. DHU-Net demonstrates improved robustness and shape integrity, although residual boundary errors and artifacts persist. CDEIT produces visually sharper reconstructions with enhanced contrast; however, incorrect shape predictions are observed in some samples, indicating that voltage-only conditioning may be insufficient to fully constrain the solution space. In contrast, MS-CDM consistently generates reconstructions that are closest to the ground truth across all tested cases. It accurately preserves target locations, geometric shapes, and boundary continuity, even in highly complex multiphase scenarios. These qualitative observations are in strong agreement with the improvements observed in Dice, SSIM, and CC metrics.

### 5.2. Ablation Study

To further investigate the contribution of different conditional constraints in the proposed MS-CDM framework, systematic ablation experiments were conducted on the same simulated dataset under identical experimental settings. By selectively removing or isolating specific conditioning information, three model variants were constructed for comparison:

(1) DC-CDM (Data-Constrained CDM): a diffusion model conditioned solely on boundary voltage measurements, designed to evaluate the effect of data-driven constraints;
(2) PG-CDM (Physics-Guided CDM): a diffusion model conditioned only on Gauss–Newton one-step reconstruction images, aimed at assessing the role of physics-guided structural priors;
(3) MS-CDM (Multi-Source CDM): the full model integrating both boundary voltage constraints and physics-guided priors.

**Figure 8:**
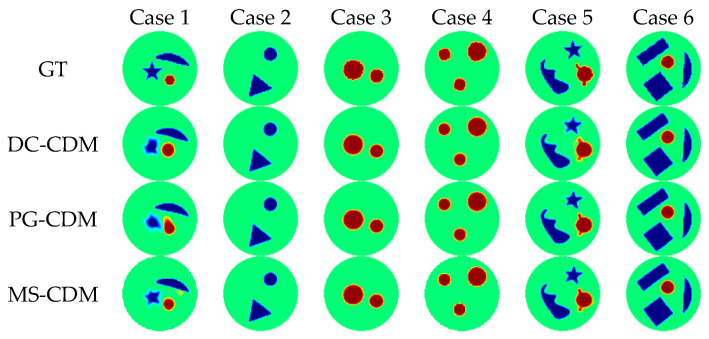
Qualitative comparison of reconstruction results obtained by different conditional diffusion model variants in the ablation study on the simulated dataset. Each column corresponds to one test sample. The first row shows the ground-truth conductivity distributions (GT), followed by the reconstructions produced by DC-CDM, PG-CDM, and the proposed MS-CDM, respectively.

Figure 8 presents representative reconstruction results from six typical test samples obtained using the three model variants. When conditioned only on boundary voltages, DC-CDM is able to recover the overall distribution of conductivity changes; however, blurred boundaries and local shape distortions remain evident in complex scenarios, indicating that data constraints alone are insufficient to fully regularize the ill-posed inverse problem. In contrast, PG-CDM produces more stable reconstructions in terms of target localization and global geometry, with clearer contours and more reasonable topological structures. This observation confirms that physics-guided priors provide effective spatial constraints that reduce the solution space and enhance structural consistency, although some local details may still appear over-smoothed. By jointly incorporating both data-driven and physics-guided conditions, MS-CDM consistently achieves the highest reconstruction quality across all test cases. Its reconstructions closely match the ground truth in terms of target position, shape integrity, and boundary continuity, and exhibit notably improved robustness in multi-target and complex geometric scenarios. These qualitative results demonstrate the complementary nature of multi-source conditioning in guiding the diffusion process.

Quantitative comparisons on the simulated dataset are summarized in Table 2. A clear and consistent performance improvement is observed as the conditioning information is progressively enriched. DC-CDM achieves a relative error of 0.256 ± 0.124 and a Dice coefficient of 0.940 ± 0.062, indicating reasonable numerical accuracy but limited structural consistency. With the introduction of physics-guided priors, PG-CDM outperforms DC-CDM across all metrics, reducing the relative error to 0.242 ± 0.110 while simultaneously improving SSIM and Dice scores, thereby validating the stabilizing effect of physical guidance. The proposed MS-CDM delivers the best overall performance on all evaluation metrics. Compared with DC-CDM and PG-CDM, its relative error is reduced by approximately 14.8% and 9.9%, respectively, while CC, SSIM, and Dice reach the highest values with the smallest standard deviations. This indicates not only higher reconstruction accuracy but also improved consistency across different samples.

**Figure 9:**
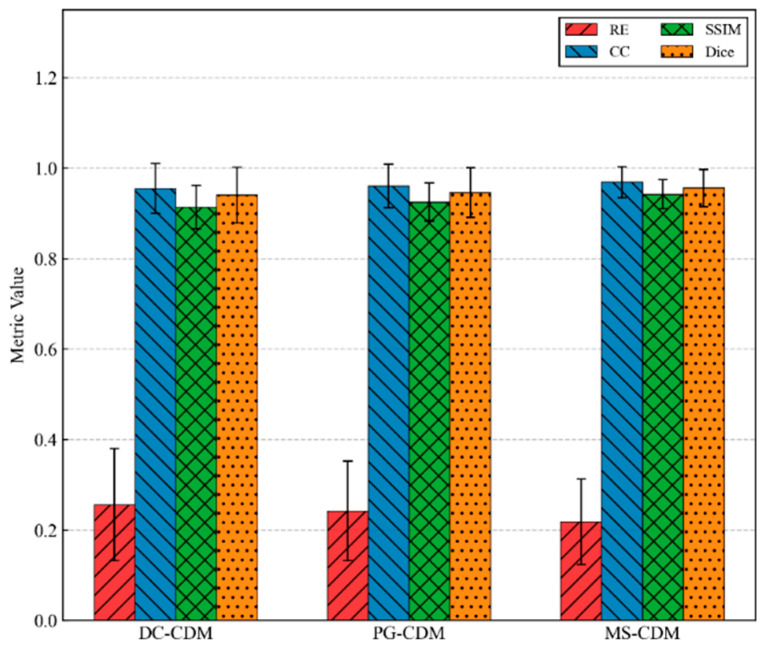
Quantitative comparison of different conditional diffusion model variants in the ablation study using four evaluation metrics.

Figure 9 further illustrates these trends using bar charts, clearly highlighting the overall superiority of MS-CDM across all evaluation metrics. The ablation results demonstrate that while single-source conditioning can partially improve reconstruction performance, only the synergistic integration of data constraints and physics-guided priors enables diffusion models to simultaneously achieve high numerical accuracy, structural fidelity, and reconstruction stability in challenging EIT inverse problems. These findings provide strong experimental evidence supporting the necessity and effectiveness of the proposed dual-conditioning design in MS-CDM.

The sensitivity of MS-CDM to the physics-guided prior was additionally examined by varying the GN regularization strength (NOSER prior, λ) and by testing alternative reconstruction priors. The results showed only limited variation in quantitative metrics, while the overall reconstruction advantage of MS-CDM remained consistent, indicating that the proposed framework is not overly sensitive to moderate changes in prior quality/type.

### 5.3. Noise Robustness Evaluation

In practical EIT measurements, boundary voltage signals are inevitably corrupted by electronic noise, electrode contact impedance fluctuations, and environmental disturbances. To systematically evaluate the robustness and stability of the proposed MS-CDM under different noise conditions, a series of noise robustness experiments was conducted on the simulated dataset by injecting multi-level Gaussian white noise into the boundary voltage measurements.

**Figure 10:**
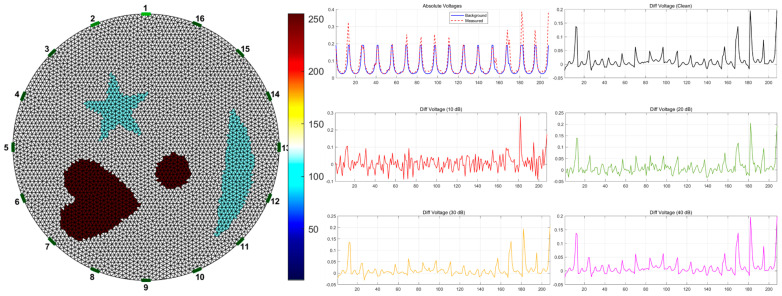
Quantitative performance comparison of the proposed MS-CDM under different SNR levels on the simulated dataset.

Five signal-to-noise ratio (SNR) scenarios were considered, including 10 dB, 20 dB, 30 dB, 40 dB, and Inf (noise-free), while keeping the network architecture, training strategy, and all other experimental settings unchanged, as illustrated in Figure 10. For fair comparison, all test samples shared identical ground-truth conductivity distributions, and noise was added exclusively at the measurement level, ensuring that performance variations could be directly attributed to changes in noise intensity.

Table 3 summarizes the quantitative reconstruction performance of MS-CDM under different SNR levels. As expected, all evaluation metrics exhibit gradual performance degradation as the SNR decreases; however, the degradation process remains smooth and controlled, without abrupt performance collapse. Under high-SNR conditions (40 dB and Inf), MS-CDM achieves highly consistent reconstruction performance. The relative error remains low (0.231 ± 0.098 and 0.218 ± 0.094, respectively), while CC, SSIM, and Dice values show negligible differences, indicating strong stability in low-noise environments. When the noise level increases to moderate ranges (30 dB and 20 dB), a noticeable yet limited performance decline is observed. Notably, at 20 dB, the Dice coefficient still reaches 0.867 ± 0.120, demonstrating that MS-CDM retains a reliable structural recovery capability under moderate noise interference. In the extreme low-SNR scenario (10 dB), reconstruction difficulty increases substantially, with the relative error rising to 0.632 ± 0.225 and corresponding reductions in CC and Dice. Nevertheless, MS-CDM maintains basic target localization ability without complete reconstruction failure, reflecting a certain degree of robustness even under severe noise conditions.

**Figure 11:**
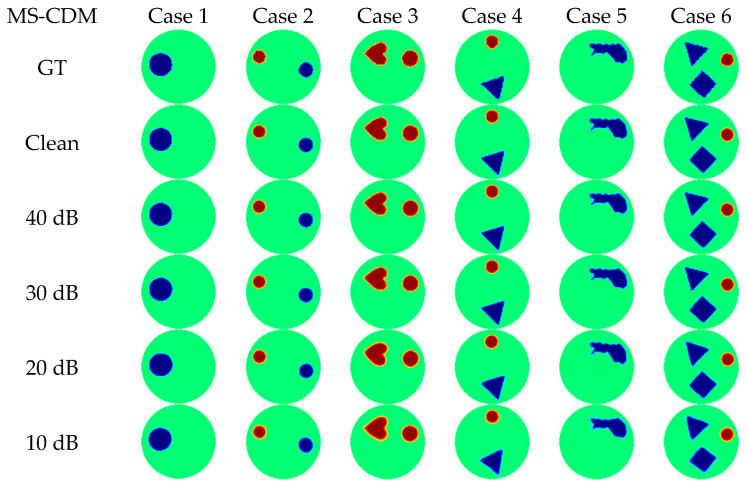
Qualitative reconstruction results of MS-CDM under different SNR levels on the simulated dataset. Each column corresponds to one test sample, and each row represents a different noise condition.

Figure 11 presents qualitative reconstruction results of MS-CDM for six representative test cases across different noise levels. Under noise-free and high-SNR conditions, the reconstructed conductivity distributions closely match the ground truth in terms of target location, geometry, and boundary continuity. As the SNR decreases to 30 dB and 20 dB, mild boundary blurring and local intensity fluctuations emerge, while the overall geometric structures remain largely preserved. Even at 10 dB, although boundary details are partially degraded and contrast is reduced in some regions, the global topological structures remain identifiable.

**Figure 12:**
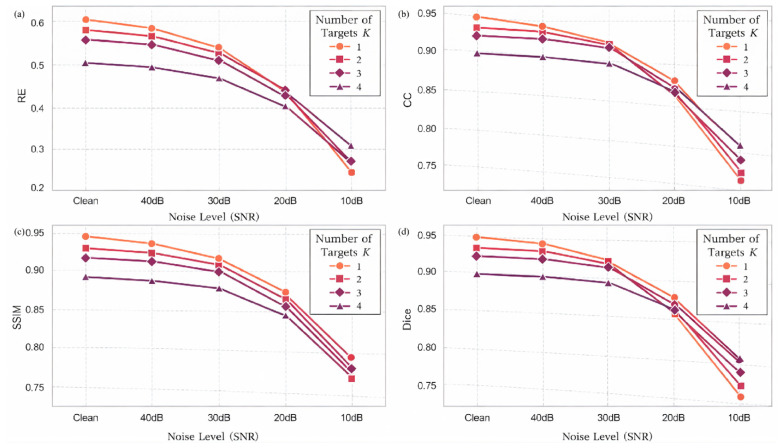
Performance trends of MS-CDM with respect to noise level: (**a**) variation in relative error (RE) across different SNR conditions; (**b**) variation in correlation coefficient (CC) across different SNR conditions; (**c**) variation in structural similarity index (SSIM) across different SNR conditions; (**d**) variation in Dice similarity coefficient across different SNR conditions.

Figure 12 further illustrates the statistical variation in all evaluation metrics with respect to SNR. The relative error exhibits a monotonic increase as noise intensifies, whereas CC, SSIM, and Dice gradually decrease. Importantly, all curves remain smooth without abrupt oscillations, indicating predictable and stable degradation behavior.

Overall, the noise robustness experiments demonstrate that MS-CDM maintains stable reconstruction performance across a wide range of noise levels, particularly excelling under moderate-to-high SNR conditions. Even under severe noise interference, the proposed model preserves essential structural information, underscoring its robustness and practical applicability in real-world EIT measurement scenarios.

## 6. Experimental Results and Analysis

To further validate the practical applicability and cross-system generalization capability of the proposed MS-CDM under real measurement conditions, extensive experiments were conducted on three representative real-world EIT datasets: the public UEF2017 dataset, the KTC2023 multi-system experimental dataset, and a self-built water tank dataset (OUR2026). These datasets exhibit substantial differences in hardware configuration, excitation protocols, electrode layouts, and noise characteristics, thereby providing a rigorous benchmark for evaluating the robustness and adaptability of EIT reconstruction algorithms across heterogeneous systems.

### 6.1. Results on the UEF2017 Dataset

**Figure 13:**
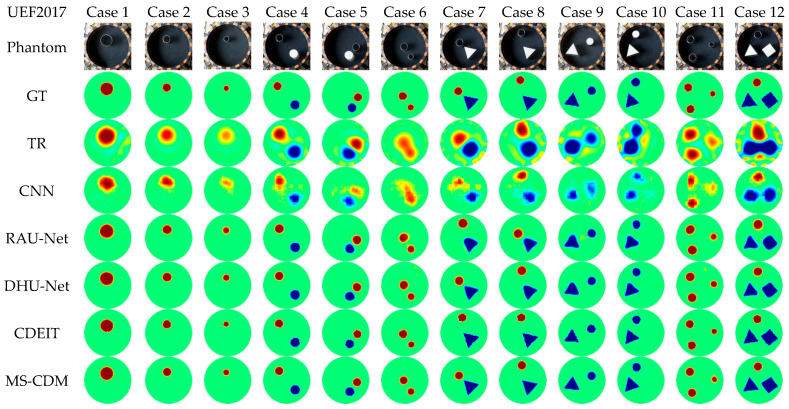
Qualitative comparison of reconstruction results obtained by different EIT methods on the UEF2017 real experimental dataset. Each column corresponds to one test sample, with the first row showing the reference conductivity distribution and the remaining rows representing different reconstruction methods.

The UEF2017 dataset is a widely used public EIT benchmark characterized by stable measurement conditions and relatively clear target structures, while still containing practical measurement noise and system imperfections. Figure 13 presents qualitative reconstruction results for 12 representative test cases, and the corresponding quantitative metrics are summarized in Table 4.

From the quantitative evaluation, the traditional TR method yields the poorest performance, suffering from severe blurring and pronounced artifacts. The CNN-based approach reduces numerical error to some extent but achieves only limited improvement in structural consistency. RAU-Net and DHU-Net exhibit noticeable gains in CC, SSIM, and Dice metrics, indicating that residual connections and dual-branch designs are beneficial for enhancing structural recovery under real measurement conditions.

**Figure 14:**
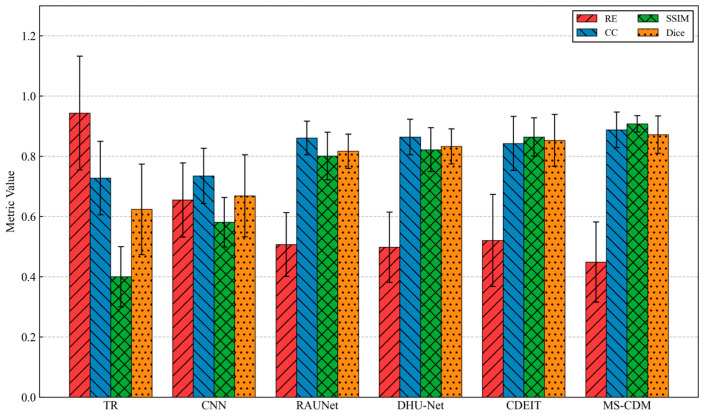
Quantitative comparison of different EIT reconstruction methods on the UEF2017 dataset in terms of RE, CC, SSIM, and Dice.

In contrast, the proposed MS-CDM consistently achieves the best performance across all evaluation metrics. Specifically, MS-CDM attains an SSIM of 0.908 ± 0.027 and a Dice coefficient of 0.872 ± 0.062, outperforming all competing methods. Qualitative comparisons further demonstrate that MS-CDM produces sharper boundaries and higher spatial resolution, enabling more accurate recovery of target morphology and location, as shown in Figure 14. These results confirm that, even in real experimental settings, the proposed multi-source conditional diffusion framework effectively suppresses measurement noise while preserving stable and physically plausible structural reconstructions.

### 6.2. Results on the KTC2023 Dataset

**Figure 15:**
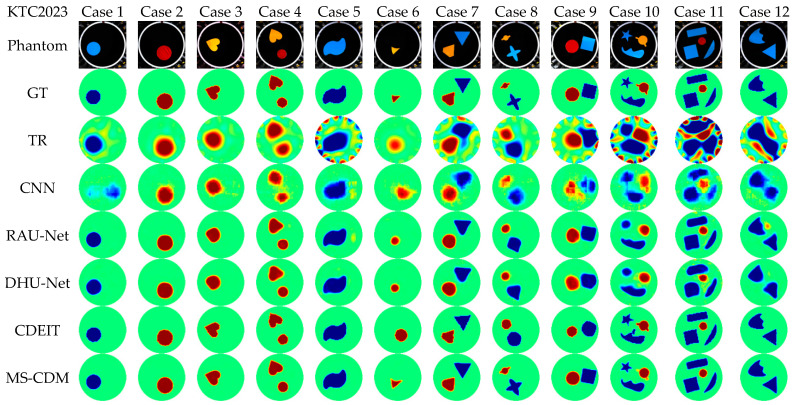
Qualitative comparison of reconstruction results obtained by different EIT methods on the KTC2023 multi-system real experimental dataset.

The KTC2023 dataset was collected using multiple EIT systems with substantially different hardware configurations, excitation protocols, and electrode arrangements. Compared with UEF2017, this dataset exhibits higher noise levels and more pronounced measurement nonidealities, thereby imposing stricter requirements on the generalization capability of reconstruction models. Figure 15 presents qualitative reconstruction results for 12 representative test cases, while the corresponding quantitative metrics are summarized in Table 5.

On this dataset, the performance of traditional methods and several supervised learning models degrades noticeably. In particular, the TR and CNN approaches suffer from severe numerical deviations and structural distortions, reflecting their limited robustness under strong system mismatch and noise contamination. RAU-Net and DHU-Net improve structural consistency to some extent; however, boundary blurring and shape instability remain evident in more complex scenarios.

**Figure 16:**
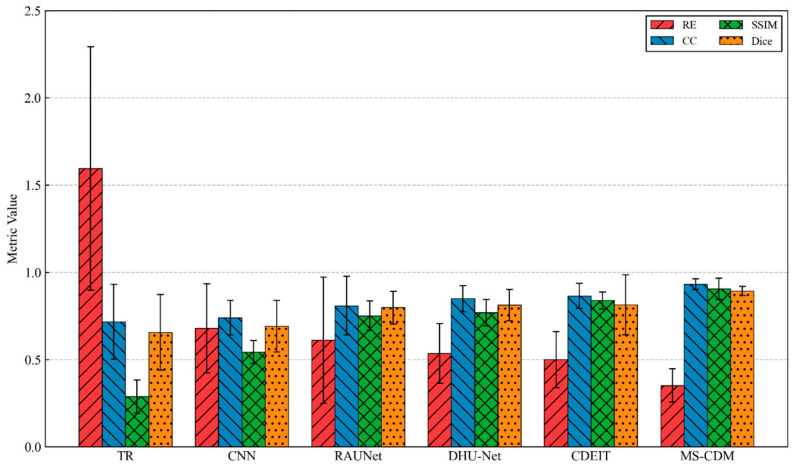
Quantitative comparison of different EIT reconstruction methods on the KTC2023 dataset in terms of RE, CC, SSIM, and Dice.

In contrast, the proposed MS-CDM demonstrates a pronounced advantage on the KTC2023 dataset. The relative error is significantly reduced to 0.351 ± 0.095, while the CC reaches 0.932 ± 0.031, with SSIM and Dice also achieving the highest values among all compared methods. Qualitative results further indicate that MS-CDM maintains high spatial resolution and stable morphology recovery even under high-noise and cross-system conditions, as shown in Figure 16. These findings clearly validate the robustness and strong cross-system generalization capability of the proposed multi-source conditional diffusion framework in realistic and heterogeneous EIT experimental environments.

### 6.3. Results on the Self-Built Water Tank Dataset

To further assess the practical applicability of the proposed MS-CDM in realistic engineering environments, a self-built water tank EIT experimental platform (OUR2026) was developed, and multiple sets of real measurement data were collected.

**Figure 17:**
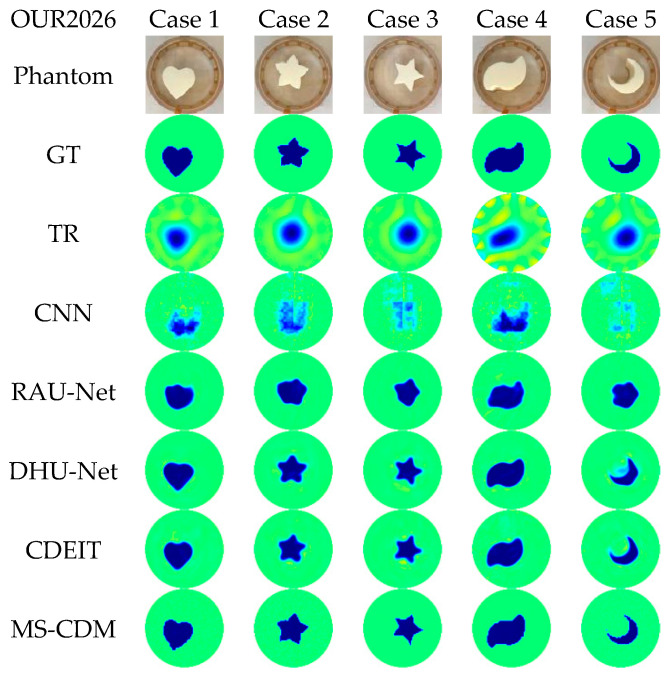
Qualitative comparison of reconstruction results obtained by different EIT methods on the self-built water tank real experimental dataset (OUR2026).

As shown in Figure 17 and summarized quantitatively in Table 6, MS-CDM again achieves the best overall performance on the OUR2026 dataset. In terms of numerical accuracy, MS-CDM attains the lowest relative error (RE = 0.358 ± 0.122), outperforming the strongest competing methods DHU-Net (0.403 ± 0.171) and CDEIT (0.384 ± 0.153). Meanwhile, MS-CDM also achieves the highest correlation coefficient (CC = 0.915 ± 0.057), indicating more accurate recovery of target locations and global spatial distributions under real measurement conditions.

With respect to structural fidelity, MS-CDM consistently yields superior performance, reaching an SSIM of 0.907 ± 0.023 and a Dice coefficient of 0.901 ± 0.055, both of which are higher than those of DHU-Net and CDEIT. These improvements demonstrate that the proposed method better preserves boundary continuity and target morphology, even in the presence of contact impedance variations and environmental disturbances. Moreover, the relatively smaller standard deviations across all metrics indicate improved reconstruction stability across different test samples.

**Figure 18:**
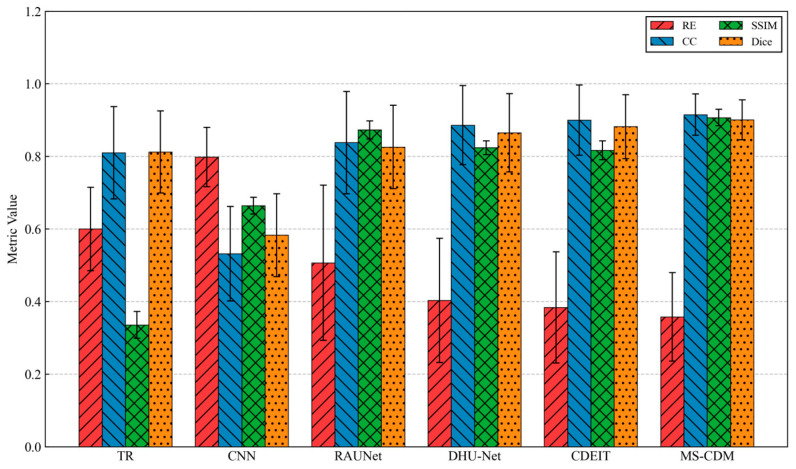
Quantitative comparison of different EIT reconstruction methods on the OUR2026 dataset in terms of RE, CC, SSIM, and Dice.

Qualitative comparisons in Figure 18 further corroborate the quantitative findings. While DHU-Net and CDEIT produce visually plausible reconstructions, residual artifacts and boundary discontinuities are still observable in some cases. In contrast, MS-CDM effectively suppresses artifacts induced by measurement noise and system nonidealities, generating clearer, more continuous target boundaries and more consistent conductivity distributions.

Collectively, these results confirm that the proposed MS-CDM maintains robust and reliable reconstruction performance under realistic experimental conditions, highlighting its strong potential for deployment in practical EIT systems.

## 7. Conclusions

This paper presented a Physics-Guided and Data-Constrained Multi-Source Conditional Diffusion Model (MS-CDM) for EIT image reconstruction. By jointly incorporating boundary voltage measurements as data-driven constraints and Gauss–Newton reconstruction images as physics-guided priors, the proposed framework effectively addresses the severe ill-posedness and instability inherent in the EIT inverse problem.

Beyond achieving high reconstruction accuracy on simulated datasets, the proposed method was extensively validated on three representative real-world EIT experimental datasets, including the public UEF2017 dataset, the multi-system KTC2023 dataset, and a self-built water tank platform (OUR2026). The consistent performance improvements across these heterogeneous datasets demonstrate the practical applicability and cross-system transferability of MS-CDM under realistic measurement conditions. Comprehensive experimental results lead to the following conclusions.

First, in terms of spatial resolution, MS-CDM is able to more clearly recover target boundaries and geometric structures, significantly outperforming conventional numerical reconstruction methods and supervised deep learning models. Second, in terms of noise robustness, MS-CDM maintains stable reconstruction performance across a wide range of noise levels and complex measurement environments, exhibiting controlled and predictable performance degradation without catastrophic failure. Third, in terms of cross-system applicability, MS-CDM consistently produces reliable reconstruction results on EIT systems with different hardware configurations and excitation protocols without requiring system-specific retraining under the studied evaluation setting.

These advantages primarily stem from the proposed multi-source conditional diffusion framework, in which data consistency and physical consistency are jointly enforced throughout the reverse denoising process. By continuously constraining the solution space with complementary measurement-domain and physics-guided priors, MS-CDM effectively reduces ambiguity in the inverse problem and achieves accurate, stable, and transferable EIT image reconstruction under practical conditions.

At the same time, several limitations should be noted. First, for real experimental phantom data, true pixel-wise internal conductivity ground truth is not directly available; therefore, quantitative metrics on real data are based on reference maps and should be interpreted primarily as comparative consistency measures rather than absolute physical accuracy. Second, although strong performance is observed across multiple EIT systems, the current cross-system validation is conducted within the protocol variations represented in the training simulations, and generalization to entirely unseen hardware protocols/geometries still requires further investigation. Third, the robustness of MS-CDM to prior quality is effective under moderate variations, but severely degraded conventional reconstruction priors may weaken structural guidance and reduce final reconstruction quality. Finally, due to iterative diffusion sampling, the computational cost remains higher than that of conventional one-step and feed-forward supervised methods, and further acceleration is needed for high-frame-rate deployment.

Future work will focus on extending the proposed framework to three-dimensional EIT imaging and dynamic reconstruction, while also improving inference efficiency (e.g., accelerated sampling and lightweight deployment) to better support time-sensitive clinical monitoring and industrial process imaging applications.

## Figures and Tables

**Table 1:** Quantitative comparison of reconstruction performance on the simulated dataset (mean ± standard deviation). The best results are highlighted in bold.

	TR	CNN	RAU-Net	DHU-Net	CDEIT	MS-CDM
RE	0.952 ± 0.303	0.564 ± 0.108	0.462 ± 0.214	0.337 ± 0.110	0.298 ± 0.102	**0.218 ± 0.094**
CC	0.770 ± 0.087	0.803 ± 0.077	0.862 ± 0.144	0.929 ± 0.049	0.944 ± 0.043	**0.969 ± 0.034**
SSIM	0.414 ± 0.126	0.590 ± 0.070	0.885 ± 0.058	0.864 ± 0.057	0.898 ± 0.049	**0.942 ± 0.032**
Dice	0.690 ± 0.152	0.756 ± 0.100	0.871 ± 0.139	0.901 ± 0.060	0.918 ± 0.052	**0.956 ± 0.041**

**Table 2:** Quantitative ablation results of different conditional diffusion model variants on the simulated dataset (mean ± standard deviation). The best results are highlighted in bold.

	DC-CDM	PG-CDM	MS-CDM
RE	0.256 ± 0.124	0.242 ± 0.110	**0.218 ± 0.094**
CC	0.955 ± 0.055	0.960 ± 0.048	**0.969 ± 0.034**
SSIM	0.914 ± 0.048	0.925 ± 0.042	**0.942 ± 0.032**
Dice	0.940 ± 0.062	0.946 ± 0.055	**0.956 ± 0.041**

**Table 3:** Quantitative reconstruction performance of MS-CDM under different SNR conditions on the simulated dataset (mean ± standard deviation).

	10 dB	20 dB	30 dB	40 dB	Inf
RE	0.632 ± 0.225	0.406 ± 0.170	0.279 ± 0.117	0.231 ± 0.098	0.218 ± 0.094
CC	0.730 ± 0.206	0.888 ± 0.114	0.949 ± 0.051	0.965 ± 0.036	0.969 ± 0.034
SSIM	0.794 ± 0.053	0.890 ± 0.043	0.930 ± 0.034	0.941 ± 0.032	0.942 ± 0.032
Dice	0.706 ± 0.203	0.867 ± 0.120	0.933 ± 0.061	0.951 ± 0.045	0.956 ± 0.041

**Table 4:** Quantitative performance comparison of different EIT reconstruction methods on the UEF2017 real experimental dataset (mean ± standard deviation). The best results are highlighted in bold.

	TR	CNN	RAUNet	DHU-Net	CDEIT	MS-CDM
RE	0.944 ± 0.189	0.655 ± 0.123	0.507 ± 0.106	0.498 ± 0.117	0.521 ± 0.153	**0.449 ± 0.133**
CC	0.728 ± 0.122	0.735 ± 0.092	0.861 ± 0.056	0.864 ± 0.059	0.843 ± 0.090	**0.888 ± 0.059**
SSIM	0.400 ± 0.100	0.581 ± 0.082	0.801 ± 0.079	0.822 ± 0.073	0.864 ± 0.064	**0.908 ± 0.027**
Dice	0.624 ± 0.150	0.669 ± 0.136	0.817 ± 0.057	0.833 ± 0.058	0.853 ± 0.086	**0.872 ± 0.062**

**Table 5:** Quantitative performance comparison of different EIT reconstruction methods on the KTC2023 real experimental dataset (mean ± standard deviation). The best results are highlighted in bold.

	TR	CNN	RAUNet	DHU-Net	CDEIT	MS-CDM
RE	1.594 ± 0.697	0.679 ± 0.255	0.611 ± 0.361	0.535 ± 0.171	0.499 ± 0.161	**0.351 ± 0.095**
CC	0.717 ± 0.214	0.739 ± 0.099	0.808 ± 0.168	0.849 ± 0.075	0.865 ± 0.072	**0.932 ± 0.031**
SSIM	0.288 ± 0.095	0.543 ± 0.066	0.751 ± 0.085	0.769 ± 0.076	0.838 ± 0.049	**0.905 ± 0.060**
Dice	0.656 ± 0.217	0.691 ± 0.148	0.798 ± 0.094	0.812 ± 0.090	0.814 ± 0.172	**0.893 ± 0.026**

**Table 6:** Quantitative performance comparison of different EIT reconstruction methods on the self-built water tank real experimental dataset (mean ± standard deviation).

	TR	CNN	RAUNet	DHU-Net	CDEIT	MS-CDM
RE	0.600 ± 0.115	0.798 ± 0.082	0.507 ± 0.214	0.403 ± 0.171	0.384 ± 0.153	0.358 ± 0.122
CC	0.810 ± 0.127	0.532 ± 0.130	0.838 ± 0.141	0.886 ± 0.109	0.900 ± 0.097	0.915 ± 0.057
SSIM	0.336 ± 0.037	0.664 ± 0.023	0.873 ± 0.025	0.824 ± 0.019	0.817 ± 0.026	0.907 ± 0.023
Dice	0.812 ± 0.113	0.583 ± 0.114	0.826 ± 0.115	0.865 ± 0.108	0.882 ± 0.088	0.901 ± 0.055

## Data Availability

Available upon request.

## References

[1] Qu S., Feng E., Dong D., Yang L., Dai M., Frerichs I., Liu S., Gao Y., Zheng J., Song L. (2025). Early screening of lung function by electrical impedance tomography in people with normal spirometry reveals unrecognized pathological features. Nat. Commun..

[2] Wu Y., Yu Y., Tian H., Li Z., Wang H., Liu K., Yao J. (2025). Spatiotemporal residual recurrent neural network for lung function evaluation using electrical impedance tomography. IEEE Trans. Instrum. Meas..

[3] Li Z., Wu Y., Liu K., Zhang Y., Chen B., Wang H., Yao J. (2026). DSFNet: Dual-source and spatiotemporal-feature fusion network for bedside diagnosis of lung injuries with electrical impedance tomography. Med. Image Anal..

[4] Chen H., Ren J., Wu Y., Liu K., Tian H., Wang H., Yao J. (2025). Rapid etiological visualization of pleural effusion with bioimpedance spectroscopy tomography. Measurement.

[5] Zhang Y., Li S., He J., Wu Y., Wang H., Liu K., Yao J. (2025). Regional Identification of Breast Tumors Using Multichannel Bioimpedance Spectroscopy. IEEE Sens. J..

[6] Zhu Z., Li G., Luo M., Zhang P., Gao Z. (2023). Electrical Impedance Tomography of Industrial Two-Phase Flow based on radial basis function neural network optimized by the Artificial Bee colony algorithm. Sensors.

[7] Kim K., Hong J.-H., Bae K., Lee K., Lee D.J., Park J., Zhang H., Sang M., Ju J.E., Cho Y.U. (2024). Extremely durable electrical impedance tomography–based soft and ultrathin wearable e-skin for three-dimensional tactile interfaces. Sci. Adv..

[8] Peng T., Li G., Li Z., Wu Y., Liu K., Yao J. (2025). An intelligent control strategy for high-flow nasal cannula based on electrical impedance tomography. Rev. Sci. Instrum..

[9] Adler A., Boyle A. (2017). Electrical impedance tomography: Tissue properties to image measures. IEEE Trans. Biomed. Eng..

[10] Santosa F., Vogelius M. (1990). A backprojection algorithm for electrical impedance imaging. SIAM J. Appl. Math..

[11] Vauhkonen M., Vadasz D., Karjalainen P.A., Somersalo E., Kaipio J.P. (1998). Tikhonov regularization and prior information in electrical impedance tomography. IEEE Trans. Med. Imaging.

[12] Xu Y., Pei Y., Dong F. (2016). An adaptive Tikhonov regularization parameter choice method for electrical resistance tomography. Flow Meas. Instrum..

[13] Zhou Z., dos Santos G.S., Dowrick T., Avery J., Sun Z., Xu H., Holder D.S. (2015). Comparison of total variation algorithms for electrical impedance tomography. Physiol. Meas..

[14] Wu Y., Chen B., Liu K., Huang S., Li Y., Jia J., Yao J. (2022). Bayesian image reconstruction using weighted Laplace prior for lung respiratory monitoring with electrical impedance tomography. IEEE Trans. Instrum. Meas..

[15] Zhang T., Tian X., Liu X., Ye J., Fu F., Shi X., Liu R., Xu C. (2022). Advances of deep learning in electrical impedance tomography image reconstruction. Front. Bioeng. Biotechnol..

[16] Dimas C., Alimisis V., Uzunoglu N., Sotiriadis P.P. (2024). Advances in electrical impedance tomography inverse problem solution methods: From traditional regularization to deep learning. IEEE Access.

[17] Zhang H., Wang Q., Li N. (2024). DA-net: A dense attention reconstruction network for lung electrical impedance tomography (EIT). IEEE Internet Things J..

[18] Tan C., Lv S., Dong F., Takei M. (2018). Image reconstruction based on convolutional neural network for electrical resistance tomography. IEEE Sens. J..

[19] Hamilton S.J., Hauptmann A. (2018). Deep D-bar: Real-time electrical impedance tomography imaging with deep neural networks. IEEE Trans. Med. Imaging.

[20] Wei Z., Liu D., Chen X. (2019). Dominant-current deep learning scheme for electrical impedance tomography. IEEE Trans. Biomed. Eng..

[21] Chen Z., Yang Y. (2021). Structure-aware dual-branch network for electrical impedance tomography in cell culture imaging. IEEE Trans. Instrum. Meas..

[22] Li X., Zhang R., Wang Q., Duan X., Sun Y., Wang J. (2023). SAR-CGAN: Improved generative adversarial network for EIT reconstruction of lung diseases. Biomed. Signal Process. Control.

[23] Li Z., Wu Y., Qi C., Wang H., Xie Y., Wu H., Tian H., Liu K., Yao J. (2025). RAU-Net3D: A two-stage deep learning method for pneumothorax volume assessment with three-dimensional electrical impedance tomography. Measurement.

[24] Jin B., Rundell W. (2015). A tutorial on inverse problems for anomalous diffusion processes. Inverse Probl..

[25] Yang X., Zhang Y., Chen H., Ma G., Wang X. (2025). A Physics-Embedded Dual-Learning Imaging Framework for Electrical Impedance Tomography. Neural Netw..

[26] Liu J., Shi F., Xiong H., Zhou Y. (2024). DiffusionEIT: Diffusion Model for Electrical Impedance Tomography. IEEE Trans. Instrum. Meas..

[27] Shi S., Kang R., Liatsis P. (2025). A conditional diffusion model for electrical impedance tomography image reconstruction. IEEE Trans. Instrum. Meas..

[28] Darbas M., Heleine J., Mendoza R., Velasco A.C. (2021). Sensitivity analysis of the complete electrode model for electrical impedance tomography. Aims Math..

[29] Pennati F., Angelucci A., Morelli L., Bardini S., Barzanti E., Cavallini F., Conelli A., Di Federico G., Paganelli C., Aliverti A. (2023). Electrical impedance tomography: From the traditional design to the novel frontier of wearables. Sensors.

[30] Wu Y., Zhang X., Yu H., Jiang C., Sun B., Yao J. (2026). Spatiotemporal Electrical Impedance Tomography for Speech Respiratory Assessment in Cleft Palate: An Interpretable Machine Learning Study. Prog. Biochem. Biophys..

[31] Jeong S., Kim H., Kim Y.H., Park C.-S., Jung H., Kim H.K. (2025). Spatiotemporal Anomaly Detection in Distributed Acoustic Sensing Using a GraphDiffusion Model. Sensors.

[32] Basak R., Wahid K.A. (2022). An In Situ Electrical Impedance Tomography Sensor System for Biomass Estimation of Tap Roots. Plants.

[33] Adler A., Lionheart W.R. (2006). Uses and abuses of EIDORS: An extensible software base for EIT. Physiol. Meas..

[34] Hauptmann A., Kolehmainen V., Mach N.M., Savolainen T., Seppänen A., Siltanen S. (2017). Open 2D electrical impedance tomography data archive. arXiv.

[35] Räsänen M., Kuusela P., Jauhiainen J., Arif M., Scheel K., Savolainen T., Seppänen A. (2023). Kuopio Tomography Challenge 2023 Open Electrical Impedance Tomographic Dataset (KTC 2023).

[36] Huang Y., Chen M., He W., Li B. (2013). A parameter selecting method of newton’s one-step error reconstructor in electrical impedance tomography imaging. J. Inf. Comput. Sci.

[37] Wu Y., Chen B., Liu K., Zhu C., Pan H., Jia J., Wu H., Yao J. (2021). Shape reconstruction with multiphase conductivity for electrical impedance tomography using improved convolutional neural network method. IEEE Sens. J..

[38] Wang Z., Li X., Sun Y., Wang Q. (2024). Electrical impedance tomography deep imaging with dual-branch U-Net based on deformable convolution and hyper-convolution. IEEE Trans. Instrum. Meas..

